# Analysis of subsequent therapy in Japanese patients with hormone receptor‒positive/human epidermal growth factor receptor 2‒negative advanced breast cancer who received palbociclib plus endocrine therapy in PALOMA-2 and -3

**DOI:** 10.1007/s12282-020-01162-4

**Published:** 2020-10-21

**Authors:** Norikazu Masuda, Hirofumi Mukai, Kenichi Inoue, Yoshiaki Rai, Shinji Ohno, Shoichiro Ohtani, Chikako Shimizu, Satoshi Hashigaki, Yasuaki Muramatsu, Yoshiko Umeyama, Hiroji Iwata, Masakazu Toi

**Affiliations:** 1 National Hospital Organization Osaka National Hospital, 2-1-14, Hoenzaka, Chuou-ku, Osaka-City, 540-0006 Japan; 2 National Cancer Center Hospital East, 6-5-1, Kashiwanoha, Kashiwa-shi, Chiba, 277-8577 Japan; 3 Saitama Cancer Center, 780, Komuro, Ina-machi, Kitaadachi-gun, Saitama, 362-0806 Japan; 4 Sagara Hospital, 3-31, Matsubara-cho, Kagoshima City, 892-0833 Japan; 5 The Cancer Institute Hospital of JFCR, 3-8-31, Ariake, Koto-ku, Tokyo, 135-8550 Japan; 6 Hiroshima City Hiroshima Citizens Hospital, 7-33, Motomachi, Naka-ku, Hiroshima, 730-8518 Japan; 7 National Center for Global Health and Medicine, 1-21-1, Toyama, Shinjuku-ku, Tokyo, 162-8655 Japan; 8 Pfizer R&D Japan, 3-22-7, Yoyogi, Shibuya-ku, Tokyo, 151-8589 Japan; 9 Pfizer Japan Inc, 3-22-7, Yoyogi, Shibuya-ku, Tokyo, 151-8589 Japan; 10 Aichi Cancer Center Hospital, 1-1, Kanokoden, Chikusa-ku, Nagoya, 464-8681 Japan; 11 Kyoto University Graduate School of Medicine, 54, Kawaharacho, Shogoin, Sakyo-ku, Kyoto, 606-8507 Japan

**Keywords:** Palbociclib, Japanese, Subsequent therapy, Advanced breast cancer

## Abstract

**Background:**

In the double-blind, phase 3 PALOMA-2 and PALOMA-3 studies, palbociclib plus endocrine therapy (ET) demonstrated significant improvement in progression-free survival versus placebo plus ET in patients with hormone receptor‒positive/human epidermal growth factor receptor 2‒negative advanced breast cancer. This analysis assessed subsequent treatment patterns after palbociclib therapy in Japanese patients enrolled in the PALOMA-2 and PALOMA-3 studies.

**Methods:**

PALOMA-2 included postmenopausal women who had not received prior systemic therapy for advanced disease. PALOMA-3 included pre- or postmenopausal women who had progressed on previous ET. Types of subsequent therapy were assessed, and treatment durations of subsequent therapy were estimated using the Kaplan–Meier method.

**Results:**

Japanese patients were enrolled in PALOMA-2 (*n* = 46) and PALOMA-3 (*n* = 35). In both studies, the most common first subsequent therapy was ET (PALOMA-2, 77% in the palbociclib group and 75% in the placebo group; PALOMA-3, 55% and 43%, respectively), followed by chemotherapy (PALOMA-2, 18% and 8%; PALOMA-3, 32% and 57%). The median (95% CI) duration of first subsequent therapy was 6.4 (2.3‒13.9) months with palbociclib plus letrozole and 6.7 (2.8‒13.0) months with placebo plus letrozole in PALOMA-2 and 3.8 (2.4‒5.7) months with palbociclib plus fulvestrant and 9.7 (1.0‒not estimable) months with placebo plus fulvestrant in PALOMA-3.

**Conclusions:**

The types of first subsequent therapy received by Japanese patients in the palbociclib plus ET and placebo plus ET groups were similar. Further evaluation of subsequent therapy data in the real-world setting is warranted considering the small sample size of this analysis.

**Electronic supplementary material:**

The online version of this article (10.1007/s12282-020-01162-4) contains supplementary material, which is available to authorized users.

## Introduction

The incidence of breast cancer in Japan has increased, and it is the fifth leading cause of cancer-related mortality among Japanese women [1]. The current National Comprehensive Cancer Network (NCCN) treatment guidelines and the Japanese Breast Cancer Society Clinical practice guidelines recommend a cyclin-dependent kinase 4/6 (CDK4/6) inhibitor in combination with endocrine therapy (ET) for the treatment of patients with hormone receptor‒positive (HR +)/human epidermal growth factor receptor 2‒negative (HER2‒) metastatic breast cancer (MBC) [2, 3]. Palbociclib is a highly selective, reversible, oral CDK4/6 inhibitor [4] that has demonstrated activity in cell line models of ET resistance and acts synergistically with antiestrogens [5]. Palbociclib was approved in September 2017 in Japan for the treatment of HR + /HER2− inoperable or recurrent breast cancer [6].

In the PALOMA-2 and PALOMA-3 clinical studies, palbociclib plus ET was effective compared with placebo plus ET in patients with HR + /HER2− advanced breast cancer [7, 8]. In PALOMA-2, palbociclib plus letrozole significantly improved median progression-free survival (PFS) compared with placebo plus letrozole (27.6 vs 14.5 months; *P* < 0.0001; data cutoff: May 31, 2017), and the time from randomization to second subsequent therapy was significantly longer with palbociclib plus letrozole versus placebo plus letrozole (38.8 vs 28.8 months; *P* < 0.005) [9]. In the PALOMA-3 trial, palbociclib plus fulvestrant significantly improved median PFS versus placebo plus fulvestrant (11.2 vs 4.6 months; *P* < 0.0001; data cutoff: October 23, 2015), and the time to the end of first subsequent therapy was significantly longer with palbociclib plus fulvestrant than with placebo plus fulvestrant (18.8 vs 14.1 months; *P* < 0.001) [10, 11]. The median duration of the immediate subsequent line of therapy was 4.9 months in the palbociclib plus fulvestrant group and 6.0 months in the placebo plus fulvestrant group [10].

Recent analyses in the overall population from PALOMA-2 and PALOMA-3 have shown that the types of subsequent therapy received by patients in the palbociclib plus ET group were similar to those received by patients in the placebo plus ET group and that PFS improvement associated with palbociclib plus ET was retained in subsequent lines of therapy [9, 10]. These findings in the overall population suggest that the treatment benefit of subsequent therapy was not compromised by palbociclib [9, 10].

Previous subgroup analyses of Japanese patients from the PALOMA-2 and PALOMA-3 studies have suggested that palbociclib plus ET is effective and well tolerated in Japanese patients with HR+/HER2− MBC [12, 13]. In PALOMA-2, the median PFS among Japanese patients was 22.2 months in the palbociclib plus letrozole group compared with 13.8 months in the placebo plus letrozole group (data cutoff: February 26, 2016) [12]. Among Japanese patients from PALOMA-3, the median PFS was 13.6 months with palbociclib plus fulvestrant and 11.2 months with placebo plus fulvestrant (data cutoff: October 23, 2015) [13].

Although current guidelines recommend a CDK4/6 inhibitor in combination with ET for the treatment of patients with HR+/HER2− MBC [2, 3], these guidelines do not provide recommendations on optimal treatment sequences or subsequent treatment options following CDK4/6 inhibitor therapy. In addition, due to differences across countries in available treatment options and health insurance systems, analysis of subsequent treatment patterns following palbociclib therapy in Japanese patients is warranted. This analysis evaluated subsequent treatment patterns after palbociclib therapy in Japanese patients enrolled in the PALOMA-2 and PALOMA-3 clinical studies to provide insights on treatment options considered following palbociclib therapy in Japan.

## Methods

### Study design and patients

This analysis included Japanese patients from the PALOMA-2 and PALOMA-3 clinical studies. Details of the PALOMA-2 and PALOMA-3 studies have been described previously [7, 8]. Briefly, PALOMA-2 was a double-blind, phase 3 study that randomly assigned patients 2:1 to palbociclib 125 mg (once daily for 3 weeks, followed by 1 week off treatment) plus letrozole 2.5 mg (every day) or to placebo plus letrozole. The study included postmenopausal women with estrogen receptor‒positive/HER2− advanced breast cancer who had not received prior systemic therapy for advanced disease. Patients who received prior treatment with any CDK4/6 inhibitor were excluded.

PALOMA-3 was a double-blind phase 3 study that randomized patients 2:1 to palbociclib 125 mg (once daily for 3 weeks, followed by 1 week off treatment) plus fulvestrant 500 mg (every 14 days for the first three injections and then every 28 days) or to placebo plus fulvestrant. Pre- or postmenopausal women with HR+/HER2− advanced breast cancer who had disease progression while receiving previous ET were included in PALOMA-3. Patients were excluded if they received prior treatment with any CDK inhibitor, fulvestrant, everolimus, or any treatment whose mechanism of action inhibits the phosphatidylinositol-3-kinase-mammalian target of rapamycin (PI3K-mTOR) pathway.

Both studies were approved by an institutional review board or ethics committee at each participating site, and all patients provided written informed consent. Both studies were conducted in accordance with the International Conference on Harmonization Good Clinical Practice guidelines and the provisions of the Declaration of Helsinki.

### Outcomes and statistical analyses

The number of patients who received first and second subsequent therapy were summarized by type of therapy, and the duration of each subsequent therapy was plotted for each patient. Median treatment durations of first and second subsequent therapies (ie, the length of time patients received the first and second subsequent therapies administered after palbociclib plus ET or placebo plus ET) were estimated based on the Kaplan–Meier method. The cutoff date was May 31, 2017 for the PALOMA-2 data, and the median follow-up duration in the overall population was 37.6 months with palbociclib plus letrozole and 37.3 months with placebo plus letrozole [9]. The cutoff date was April 13, 2018 for the PALOMA-3 data, and the median follow-up duration in the overall population was 44.8 months in both arms [10].

## Results

Demographic and baseline disease characteristics of Japanese patients enrolled in PALOMA-2 and PALOMA-3 are presented in Table 1. In the overall population in PALOMA-2 and PALOMA-3, the types of subsequent therapy received by patients in the palbociclib plus ET and placebo plus ET groups were similar (Online Resource—Supplemental Table 1) [9, 10].

**Table 1:** Japanese patients: demographics and baseline disease characteristics

Characteristics	PALOMA-2		PALOMA-3	
	PAL + LET (*n* = 32)	PBO + LET (*n* = 14)	PAL + FUL (*n* = 27)	PBO + FUL (*n* = 8)
Age, median (range), y	67 (44‒88)	61 (51‒88)	53 (36‒77)	57 (39‒79)
ECOG performance status, *n* (%)				
0	27 (84)	10 (71)	27 (100)	7 (88)
1	3 (9)	4 (29)	0	1 (13)
2	2 (6)	0	‒	‒
Visceral metastases,^a^ *n* (%)				
Yes	20 (63)	10 (71)	17 (63)	7 (88)
No	12 (38)	4 (29)	10 (37)	1 (13)
Prior lines of therapy in the context of metastatic disease, *n* (%)				
0	32 (100)	14 (100)	7 (26)^b^	3 (38)^b^
1	NA	NA	14 (52)	3 (38)
2	NA	NA	3 (11)	2 (25)
≥ 3	NA	NA	3 (11)	0
Prior chemotherapy for advanced/metastatic breast cancer, *n* (%)	NA	NA	2 (7)	1 (13)
Prior (neo) adjuvant endocrine therapy, *n* (%)	21 (66)	10 (71)	NA	NA

*ECOG* Eastern Cooperative Oncology Group; *FUL* fulvestrant; *LET* letrozole; *NA* not applicable; *PAL* palbociclib; *PBO* placebo

^a^Refers to lung (including pleura) and/or liver involvement in PALOMA-2, and refers to lung, liver, brain, pleural, or peritoneal involvement in PALOMA-3

^b^Patients who progressed while receiving or ≤ 12 mo after ending adjuvant therapy

### PALOMA-2 Japanese patients

**Fig. 1:**
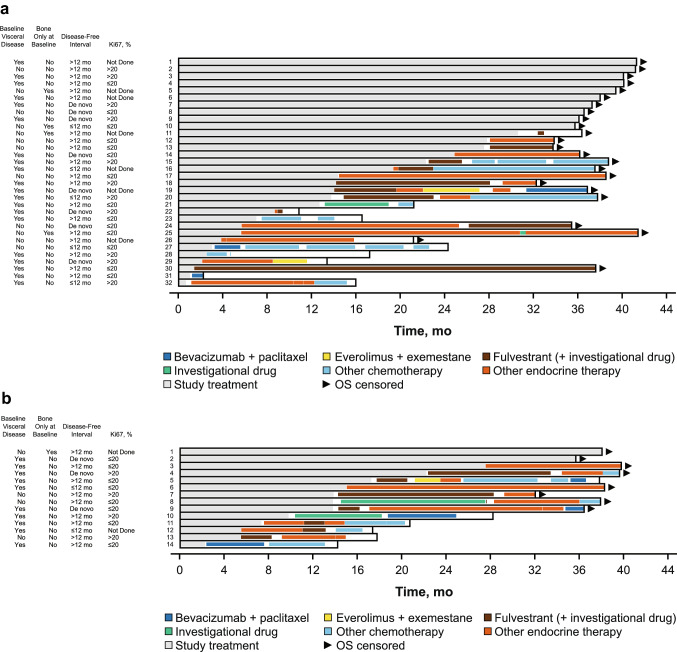
Japanese patients in PALOMA-2: swimmer plot of subsequent therapy. **a** Palbociclib group; **b** Placebo group. *OS* overall survival. Disease-free interval defined as the time between the end of (neo)adjuvant treatment and the onset of metastatic disease or disease recurrence

**Fig. 2:**
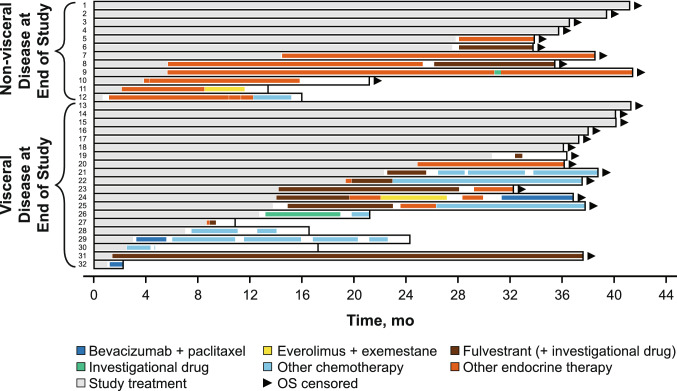
Japanese patients in PALOMA-2: swimmer plot of subsequent therapy in the palbociclib plus letrozole group by visceral disease status. *OS* overall survival

A total of 46 Japanese patients from PALOMA-2 were included in this analysis, with 32 patients in the palbociclib plus letrozole group and 14 patients in the placebo plus letrozole group. Ten patients (31%) in the palbociclib plus letrozole group and two patients (14%) in the placebo plus letrozole group were still receiving study treatment at the data cutoff date. A total of 22 (69%) patients in the palbociclib plus letrozole group and 12 patients (86%) in the placebo plus letrozole group received first subsequent therapy; among these patients, 17 (77%) and 4 (18%) patients in the palbociclib plus letrozole group and 9 (75%) and 1 (8%) patients in the placebo plus letrozole group received ET and chemotherapy, respectively, as first subsequent therapy. The types of first subsequent therapy administered in the palbociclib plus letrozole and placebo plus letrozole groups were generally similar (Table 2). None of the patients in either treatment group received everolimus plus exemestane as first subsequent therapy. The median (95% CI) duration of first subsequent therapy was also similar between the palbociclib plus letrozole (6.4 [2.3‒13.9] months) and placebo plus letrozole (6.7 [2.8‒13.0] months) groups. The treatment patterns and durations of subsequent therapies for each patient are shown in Fig. 1. All patients who did not have visceral disease at the end of the study received endocrine-based therapy following palbociclib plus letrozole treatment (Fig. 2).

**Table 2:** Japanese patients in PALOMA-2: type and duration of first and second subsequent therapy

Systemic anticancer therapy, *n* (%)	PAL + LET (*n* = 32)	PBO + LET (*n* = 14)
**No subsequent therapy received**	**10 (31)**	**2 (14)**
Study treatment ongoing	10 (100)	2 (100)
**Received first subsequent therapy**	**22 (69)**	**12 (86)**
Endocrine-based therapy	17 (77)	9 (75)
Fulvestrant	7 (32)^a^	5 (42)
Letrozole	6 (27)	2 (17)
Tamoxifen	2 (9)	1 (8)
Toremifene	1 (5)	1 (8)
Medroxyprogesterone	1 (5)	0
Chemotherapy	4 (18)	1 (8)
Bevacizumab + paclitaxel	2 (9)	1 (8)
Paclitaxel	1 (5)	0
TS-1	1 (5)	0
Other (investigational drug)	1 (5)	2 (17)
Duration of first subsequent therapy, median (95% CI), mo^b^	6.4 (2.3‒13.9)	6.7 (2.8‒13.0)
**Received second subsequent therapy**	**15 (47)**	**10 (71)**
Endocrine-based therapy	9 (60)	8 (80)
Fulvestrant	3 (20)^a^	3 (30)
Toremifene	2 (13)	1 (10)
Everolimus + exemestane	1 (7)	1 (10)
Tamoxifen	1 (7)	2 (20)
Exemestane	1 (7)	1 (10)
Anastrozole	1 (7)	0
Chemotherapy	5 (33)	2 (20)
Capecitabine	2 (13)	1 (10)
Capecitabine + fulvestrant	1 (7)	0
Docetaxel	1 (7)	0
Eribulin	1 (7)	0
Bevacizumab + paclitaxel	0	1 (10)
Other (investigational drug)	1 (7)	0
Duration of second subsequent therapy, median (95% CI), mo^b^	2.4 (0.5‒3.2)	3.7 (0.03‒6.2)

*LET* letrozole; *PAL* palbociclib; PBO placebo

^a^Including “fulvestrant” and “fulvestrant + investigational drug”

^b^Estimated using the Kaplan–Meier method

Fifteen patients (47%) in the palbociclib plus letrozole group and ten patients (71%) in the placebo plus letrozole group received second subsequent therapy. Of these, 9 (60%) and 5 (33%) patients in the palbociclib plus letrozole group and 8 (80%) and 2 (20%) patients in the placebo plus letrozole group received ET and chemotherapy, respectively, as second subsequent therapy (Table 2). In PALOMA-2, the mostly frequently used second subsequent therapy was ET. The median (95% CI) duration of second subsequent therapy was 2.4 (0.5‒3.2) months for the palbociclib plus letrozole group and 3.7 (0.03‒6.2) months for the placebo plus letrozole group.

### PALOMA-3 Japanese patients

**Fig. 3:**
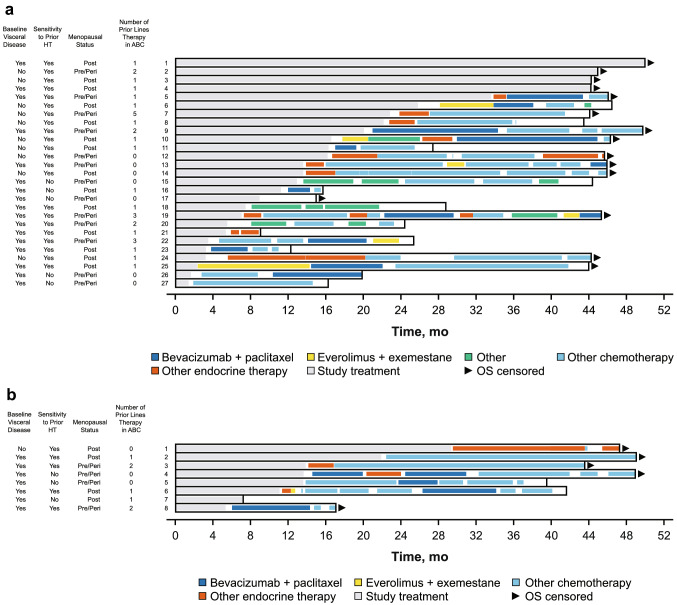
Japanese patients in PALOMA-3: swimmer plot of subsequent therapy. **a** Palbociclib group; **b** Placebo group. *ABC* advanced breast cancer; *HT* hormonal therapy; *OS* overall survival. Sensitivity to prior hormone therapy was defined as a documented clinical benefit (complete response, partial response, stable disease ≥ 24 weeks) to ≥ 1 prior hormone therapy in the metastatic setting or ≥ 24 months of adjuvant hormone therapy before recurrence

**Fig. 4:**
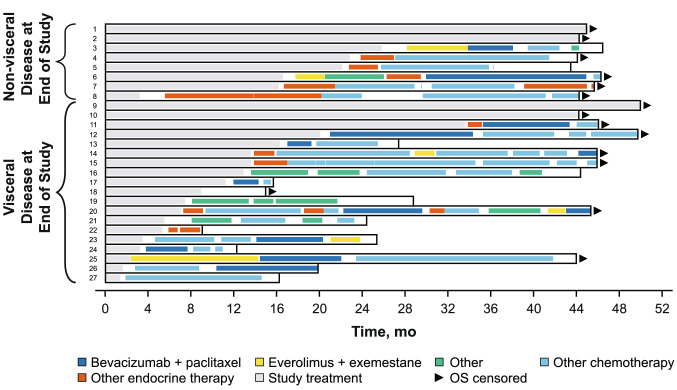
Japanese patients in PALOMA-3: swimmer plot of subsequent therapy in the palbociclib plus fulvestrant group by visceral disease status. *OS* overall survival

A total of 35 Japanese patients from PALOMA-3 were included in this analysis, with 27 patients in the palbociclib plus fulvestrant group and 8 patients in the placebo plus fulvestrant group. Four patients (15%) in the palbociclib plus fulvestrant group were still receiving study treatment at the data cutoff date. One patient in each group did not receive subsequent therapy after study treatment. Twenty-two patients (81%) in the palbociclib plus fulvestrant group and seven patients (88%) in the placebo plus fulvestrant group received first subsequent therapy; of these patients, 12 (55%) and 3 (43%) patients, respectively, received ET as first subsequent therapy (Table 3). Generally, patients in both groups had a similar type of first subsequent therapy, but the frequency of chemotherapy was slightly higher in the placebo plus fulvestrant group (4 [57%] vs 7 [32%] patients). The median (95% CI) duration of first subsequent therapy was 3.8 (2.4‒5.7) months with palbociclib plus fulvestrant and 9.7 (1.0‒not estimable) months with placebo plus fulvestrant. The treatment pattern and durations of subsequent therapies for each patient are shown in Fig. 3. Following palbociclib plus fulvestrant treatment, all patients without visceral disease at the end of the study received endocrine-based therapy (Fig. 4).

**Table 3:** Japanese patients in PALOMA-3: type and duration of first and second subsequent therapy

Systemic anticancer therapy, *n* (%)	PAL + FUL (*n* = 27)	PBO + FUL (*n* = 8)
**No subsequent therapy received**	**5 (19)**	**1 (13)**
Study treatment ongoing	4 (80)	0
Study treatment terminated	1 (20)	1 (100)
**Received first subsequent therapy**	**22 (81)**	**7 (88)**
Endocrine-based therapy	12 (55)	3 (43)
Everolimus + exemestane	3 (14)	0
Letrozole	3 (14)	0
Exemestane	2 (9)	1 (14)
Tamoxifen	2 (9)	1 (14)
Anastrozole	1 (5)	0
Anastrozole + FUL	1 (5)	0
Toremifene	0	1 (14)
Chemotherapy	7 (32)	4 (57)
Bevacizumab + paclitaxel	4 (18)	2 (29)
Capecitabine	1 (5)	1 (14)
Cyclophosphamide + doxorubicin	1 (5)	0
Eribulin	1 (5)	0
TS-1	0	1 (14)
Other (investigational drug)	3 (14)	0
Duration of first subsequent therapy, median (95% CI), mo^a^	3.8 (2.4‒5.7)	9.7 (1.0‒NE)
**Received second subsequent therapy**	**21 (78)**	**6 (75)**
Endocrine-based therapy	2 (10)	3 (50)
Toremifene	1 (5)	0
Medroxyprogesterone	1 (5)	0
Everolimus + exemestane	0	1 (17)
Exemestane	0	1 (17)
Letrozole	0	1 (17)
Chemotherapy	16 (76)	3 (50)
Bevacizumab + paclitaxel	4 (19)	1 (17)
Eribulin	4 (19)	0
TS-1	3 (14)	1 (17)
Cyclophosphamide + epirubicin	2 (10)	1 (17)
Capecitabine	2 (10)	0
Paclitaxel	1 (5)	0
Other (investigational drug and other therapy)	3 (14)	0
Duration of second subsequent therapy, median (95% CI), mo^a^	5.8 (2.8‒7.6)	3.9 (0.5‒NE)

*FUL* fulvestrant; *NE* not estimable; *PAL* palbociclib; *PBO* placebo

^a^Estimated using the Kaplan–Meier method

Of the 21 patients (78%) who received second subsequent therapy in the palbociclib plus fulvestrant group, 16 (76%) and 2 (10%) patients received chemotherapy and ET, respectively; of the 6 patients (75%) who received second subsequent therapy in the placebo plus fulvestrant group, the corresponding patient numbers and percentages were 3 (50%) and 3 (50%). Endocrine therapy more frequently used in the placebo versus palbociclib group **(**Table 3). The median (95% CI) duration of second subsequent therapy was 5.8 (2.8‒7.6) months with palbociclib plus fulvestrant and 3.9 (0.5‒not evaluable) months with placebo plus fulvestrant. An analysis of subsequent therapies by number of prior lines of therapy for advanced disease showed that 40% of patients who received at least two prior treatments before enrolling in PALOMA-3 received endocrine-based therapy following palbociclib plus fulvestrant treatment (Online Resource—Supplemental Table 2).

## Discussion

Previous subgroup analyses of Japanese patients from the PALOMA-2 and PALOMA-3 studies showed that the median PFS was 22.2 months with palbociclib plus letrozole group versus 13.8 months with placebo plus letrozole [12] and 13.6 months with palbociclib plus fulvestrant versus 11.2 months with placebo plus fulvestrant [13]. Current NCCN and Japanese Breast Cancer Society guidelines do not provide recommendations regarding optimal breast cancer treatment sequences or subsequent treatment options following CDK4/6 inhibitor therapy [2, 3]. Analyses in the overall populations from PALOMA-2 and -3 have demonstrated that the benefit gained from subsequent therapies is not affected by initial palbociclib plus ET therapy [9, 10]. However, evaluation of subsequent treatment patterns after palbociclib in Japanese patients is warranted owing to variations in available treatment options and health insurance systems across different countries.

This analysis showed that for Japanese patients in PALOMA-2 and -3, the types of first subsequent therapy administered in the palbociclib plus ET group were similar to those administered in the placebo plus ET group and were, in turn, similar to those administered in the overall study populations [9, 10]. In PALOMA-2, more than 70% of Japanese patients received endocrine-based therapy as first subsequent therapy, and fulvestrant was used in almost 30% of these patients. In PALOMA-3, approximately half of the Japanese patients received endocrine-based therapy as first subsequent therapy. Additionally, 40% of patients who received at least two prior treatments for advanced disease before enrolling in PALOMA-3 received endocrine-based therapy following palbociclib plus fulvestrant treatment. Compared with patients in PALOMA-2, chemotherapy was used more frequently in PALOMA-3. In both studies, all Japanese patients without visceral disease at the end of the study received endocrine-based therapies following palbociclib treatment. These findings, together with comprehensive knowledge about hormone-sensitivity and breast cancer disease state, suggest that physicians may prescribe endocrine-based therapy following palbociclib treatment.

The median treatment duration of first subsequent therapy in Japanese patients was similar in both treatment groups in PALOMA-2 (6.4 months in the palbociclib plus letrozole group and 6.7 months in the placebo plus letrozole group). Although comparisons between studies should be interpreted with caution and the data from previous studies included only fulvestrant, these findings are comparable with studies assessing PFS and time to treatment failure associated with fulvestrant in patients with advanced or metastatic breast cancer [14, 15]. In the Comparison of Faslodex in Recurrent or Metastatic Breast Cancer (CONFIRM) trial, fulvestrant 500 mg as second-line therapy was associated with a median PFS of 6.5 months [14]. In the Safari study, the median time to treatment failure was 6.18 months with fulvestrant 500 mg in the second-line setting [15].

In PALOMA-3, the median treatment duration of first subsequent therapy in Japanese patients differed between the palbociclib plus fulvestrant group (3.8 months) and the placebo plus fulvestrant group (9.7 months). However, these results should be evaluated with caution owing to the small number of patients who received first subsequent therapy (22 patients in the palbociclib group and seven patients in the placebo group), with 15% of patients in the palbociclib group and no patients in the placebo group still receiving ongoing study treatment. In the Japanese subgroup in PALOMA-3, because the Kaplan–Meier PFS curves of the two treatment groups crossed [13] and potential confounding factors were suspected, these confounding factors may have affected the differences in the duration of subsequent therapy as well. There was no obvious trend observed in the characteristics of patients with longer duration of subsequent hormonal therapy compared with those with shorter duration, although results were difficult to evaluate due to the small sample size (data not shown).

In Japanese patients from PALOMA-2, the median duration of second subsequent therapy was 2.4 months in the palbociclib plus letrozole group and 3.7 months in the placebo plus letrozole group; in the Japanese patients from PALOMA-3, the median duration was 5.8 months in the palbociclib plus fulvestrant group and 3.9 months in the placebo plus fulvestrant group. Given the small number of patients who received second subsequent therapy, and the observation that several patients are still receiving ongoing study treatment or first subsequent therapy, it is not possible to draw conclusions regarding whether palbociclib impacts the therapeutic effect of the second subsequent treatment.

Limitations of the current analysis include the small sample size, a relatively short duration of follow-up, particularly for PALOMA-2, and differences in the number of patients receiving ongoing study treatment in the palbociclib versus placebo groups. An additional limitation is that the clinical efficacy of the subsequent therapies patients received after palbociclib progression was not assessed.

In the overall population of patients in PALOMA-2, the median time from randomization to first subsequent therapy was longer in the palbociclib plus letrozole group compared with the placebo plus letrozole group (28.0 vs 17.7 months), as was the time to second subsequent therapy (38.8 vs 28.8 months) [9]. The 10-month improvement in PFS associated with palbociclib plus letrozole in the primary PFS analysis was preserved in both analyses, suggesting that palbociclib did not affect the benefit of the first subsequent treatment [9]. Similarly, in the overall population of patients in PALOMA-3, the median time from randomization to the end of first subsequent therapy was longer with palbociclib plus fulvestrant versus placebo plus fulvestrant (18.8 vs 14.1 months) [10]. Additionally, the duration of first subsequent therapy was similar between the palbociclib plus fulvestrant and placebo plus fulvestrant groups [10]. These findings suggest that palbociclib did not compromise the benefit of the first subsequent therapy [10]. The overall survival results for PALOMA-2 have not yet been reported, but the magnitude of PFS improvement was maintained in patients who received first subsequent therapy [9]. In PALOMA-3, palbociclib plus fulvestrant was associated with a longer time from randomization to the end of first subsequent therapy and a numerically longer median overall survival compared with placebo plus fulvestrant [10]. Together, these findings suggest that palbociclib does not compromise the benefit of the second or later subsequent therapy.

Although real-world evidence assessing subsequent treatments following palbociclib therapy is limited, a previously conducted retrospective study of 230 patients with MBC at the St. Louis Siteman Cancer Center at Washington University evaluated the patterns and outcomes of subsequent treatment following palbociclib therapy [16]. Of the 104 patients who received subsequent therapy after palbociclib, 70 received chemotherapy and 32 received hormonal therapy [16]. Median PFS for subsequent therapies after palbociclib was 4.2 months for those receiving chemotherapy and 5.6 months for those receiving hormonal therapy [16]. In patients receiving chemotherapy, median PFS was not reached after progression on first-line palbociclib treatment (*n* = 7) and 4.7 months and 4.1 months after progression on second-line (*n* = 14) and subsequent-line (*n* = 49) palbociclib treatment, respectively [16]. Median PFS was 17.0 months, 9.3 months, and 4.2 months after progression on first-line (*n* = 7), second-line (*n* = 9), and subsequent-line (*n* = 16) palbociclib treatment, respectively, among patients receiving hormonal therapy or hormonal therapy plus targeted agents [16]. Findings from the study suggested that palbociclib is effective in the real-world setting and that subsequent hormonal therapies maintain their efficacy following palbociclib in patients with MBC [16].

Future studies are warranted to further assess the efficacy of subsequent therapies and the optimal sequence of therapeutic options following palbociclib therapy in patients with MBC.

## Conclusions

In the PALOMA-2 and PALOMA-3 studies, the types of first subsequent therapy administered to Japanese patients were similar in the palbociclib plus ET and placebo plus ET groups. Generally, endocrine-based therapy may be a feasible treatment option following palbociclib, but physicians should prescribe a subsequent treatment based on the individual patient’s hormone-sensitivity status and breast cancer disease state. It is worthwhile to further evaluate subsequent therapy data in the real-world setting considering the small sample size of this analysis.

## Electronic supplementary material

Below is the link to the electronic supplementary material.

Supplementary file1 (PDF 277 kb)

## Data Availability

Upon request, and subject to certain criteria, conditions and exceptions (see https://www.pfizer.com/science/clinical-trials/trial-data-and-results for more information), Pfizer will provide access to individual de-identified participant data from Pfizer-sponsored global interventional clinical studies conducted for medicines, vaccines and medical devices (1) for indications that have been approved in the US and/or EU or (2) in programs that have been terminated (i.e., development for all indications has been discontinued). Pfizer will also consider requests for the protocol, data dictionary, and statistical analysis plan. Data may be requested from Pfizer trials 24 months after study completion. The de-identified participant data will be made available to researchers whose proposals meet the research criteria and other conditions, and for which an exception does not apply, via a secure portal. To gain access, data requestors must enter into a data access agreement with Pfizer.
