# Supplementary material for: Analysis of subsequent therapy in Japanese patients with hormone receptor‒positive/human epidermal growth factor receptor 2‒negative advanced breast cancer who received palbociclib plus endocrine therapy in PALOMA-2 and -3
Source: Breast Cancer. 2020 Oct 21;28(2):335–45. doi: 10.1007/s12282-020-01162-4 (PMC7925474; doi:10.1007/s12282-020-01162-4)
Supplement: Supplementary file 1 — Supplementary file1 (PDF 277 kb) [file 12282_2020_1162_MOESM1_ESM.pdf]

Electronic Supplementary Material

Title: Analysis of Subsequent Therapy in Japanese Patients With Hormone-Receptor–Positive/Human Epidermal Growth Factor Receptor 2–Negative Advanced Breast Cancer Who Received Palbociclib Plus Endocrine Therapy in PALOMA-2 and -3

Journal: *Breast Cancer*

Authors: Norikazu Masuda, Hirofumi Mukai, Kenichi Inoue, Yoshiaki Rai, Shinji Ohno, Shoichiro Ohtani, Chikako Shimizu, Satoshi Hashigaki, Yasuaki Muramatsu, Yoshiko Umeyama, Hiroji Iwata, Masakazu Toi

Corresponding Author

Norikazu Masuda

National Hospital Organization Osaka National Hospital

2-1-14, Hoenzaka, Chuou-ku

Osaka-City 540-0006, Japan

Telephone: +81-6-6942-1331

Fax: +81-6-6946-3608

Email: nmasuda@alpha.ocn.ne.jp

Supplemental Table 1. Overall Population: Types of First Subsequent Therapy [9,10]

	PALOMA-2 (Data Cutoff: May 31, 2017)		PALOMA-3 (Data Cutoff: April 13, 2018)	
**Systemic Anticancer Therapy, n (%)**	**PAL+LET (n=444)**	**PBO+LET (n=222)**	**PAL+FUL (n=347)**	**PBO+FUL (n=174)**
Received any first subsequent therapy	227 (51)	150 (68)	248 (71)	140 (80)
Endocrine therapy	138 (61)	87 (58)	100 (40)	52 (37)
Selective estrogen receptor degraders; FUL	70 (31)	44 (29)	NR	NR
Steroidal aromatase inhibitors; exemestane	49 (22)	35 (23)	57 (23)	25 (18)
Selective estrogen receptor modulators	16 (7)	5 (3)	NR	NR
Nonsteroidal aromatase inhibitors	3 (1)	3 (2)	NR	NR
Chemotherapy	83 (37)	51 (34)	138 (56)	87 (62)
Capecitabine	28 (12)	19 (13)	66 (27)	36 (26)
Paclitaxel	29 (13)	19 (13)	31 (12)	31 (22)
Cyclophosphamide	NR	NR	13 (5)	8 (6)
Anthracyclines	14 (6)	7 (5)	NR	NR
Doxorubicin	NR	NR	12 (5)	1 (1)
mTOR inhibitor; everolimus	31 (14)	26 (17)	40 (16)	21 (15)
CDK4/6 inhibitor	0	13 (9)	6 (2)	9 (6)
Palbociclib	0	13 (9)	4 (2)	7 (5)
Ribociclib	NR	NR	1 (<1)	2 (1)
Abemaciclib	NR	NR	1 (<1)	0
Investigational drug	23 (10)	17 (11)	NR	NR

CDK4/6=cyclin-dependent kinase 4/6; FUL=fulvestrant; LET=letrozole; mTOR=mammalian target of rapamycin; NR=not reported; PAL=palbociclib; PBO=placebo.

Supplemental Table 2. Japanese Patients in PALOMA-3: Type of First Subsequent Therapy by Number of Prior Lines of Therapy for Advanced/Metastatic Disease

	PAL+FUL (n=27)			PBO+FUL (n=8)		
**Prior lines of therapy in the context of metastatic disease**	**0 or 1 (n=21)**	**2 or ≥3 (n=6)**	**Total (n=27)**	**0 or 1 (n=6)**	**2 or ≥3 (n=2)**	**Total (n=8)**
No subsequent therapy received, n (%)	4 (19)	1 (17)	5 (19)	1 (17)	0	1 (13)
Study treatment ongoing	3 (75)	1 (100)	4 (80)	0	0	0
Study treatment terminated	1 (25)	0	1 (20)	1 (100)	0	1 (100)
Received any first subsequent therapy, n (%)	17 (81)	5 (83)	22 (81)	5 (83)	2 (100)	7 (88)
Endocrine-based therapy	10 (59)	2 (40)	12 (55)	2 (40)	1 (50)	3 (43)
Chemotherapy	5 (29)	2 (40)	7 (32)	3 (60)	1 (50)	4 (57)
Other (investigational drug)	2 (12)	1 (20)	3 (14)	0	0	0

FUL=fulvestrant; PAL=palbociclib; PBO=placebo.
